# Transmission dynamics and control of Ebola virus disease (EVD): a review

**DOI:** 10.1186/s12916-014-0196-0

**Published:** 2014-10-10

**Authors:** Gerardo Chowell, Hiroshi Nishiura

**Affiliations:** School of Human Evolution and Social Change, Arizona State University, Tempe, AZ USA; Division of International Epidemiology and Population Studies, Fogarty International Center, National Institutes of Health, 31 Center Drive, MSC 2220, Bethesda, MD 20892-2220 USA; Department of Global Health Policy, Graduate School of Medicine, The University of Tokyo, Hongo 7-3-1, Bunkyo-ku, Tokyo, 110-0033 Japan

**Keywords:** Ebola Virus Disease, Transmission model, Control interventions, Basic reproduction number, West Africa, Incubation, Serial interval, Case fatality ratio, Isolation, Behavior change

## Abstract

The complex and unprecedented Ebola epidemic ongoing in West Africa has highlighted the need to review the epidemiological characteristics of Ebola Virus Disease (EVD) as well as our current understanding of the transmission dynamics and the effect of control interventions against Ebola transmission. Here we review key epidemiological data from past Ebola outbreaks and carry out a comparative review of mathematical models of the spread and control of Ebola in the context of past outbreaks and the ongoing epidemic in West Africa. We show that mathematical modeling offers useful insights into the risk of a major epidemic of EVD and the assessment of the impact of basic public health measures on disease spread. We also discuss the critical need to collect detailed epidemiological data in real-time during the course of an ongoing epidemic, carry out further studies to estimate the effectiveness of interventions during past outbreaks and the ongoing epidemic, and develop large-scale modeling studies to study the spread and control of viral hemorrhagic fevers in the context of the highly heterogeneous economic reality of African countries.

## Background

A complex epidemic of *Zaire ebolavirus* (EBOV) has been affecting West Africa since approximately December 2013, with the first cases likely occurring in southern Guinea [1]. The causative Ebola strain is closely related to a strain associated with past EBOV outbreaks in Central Africa [2] and could have been circulating in West Africa for about a decade [2]. However, the current epidemic was not identified until March 2014 [1], which facilitated several transmission chains to progress essentially unchecked in the region and to cross porous borders with neighboring Sierra Leone and Liberia and seed a limited outbreak in Nigeria via commercial airplane on 20 July 2014 [3]. The World Health Organization declared the Ebola epidemic in West Africa a Public Health Emergency of International Concern on 8 August 2014 [4], with exponential dynamics characterizing the growth in the number of new cases in some areas [5-9]. Economic and sociocultural factors together with the delay in identifying the outbreak in urban settings have hindered a timely and effective implementation of control efforts in the region [10,11]. Remarkably, the current size of the ongoing EBOV epidemic far surpasses the total number of cases reported for all previous Ebola outbreaks combined. A total of 6,553 cases, with 3,083 deaths, have been reported to the World Health Organization as of 23 September 2014.

A serious shortage of timely resources in the region is the key factor responsible for the onset and disproportionate scale of the ongoing epidemic in West Africa [11]. In particular, the epidemic is unfolding in a region characterized by limited public health infrastructure including: (1) a lack of essential supplies to implement infection control measures in health care settings; (2) scarcity of health care workers and staff to manage a growing case burden and carry out essential contact tracing activities to find new cases quickly so that these can be effectively isolated [12]; and (3) the absence of epidemiological surveillance for the timely identification of case clusters [13,14]. Containing the ongoing epidemic poses an unprecedented challenge as the virus has moved from Guinea to reach urban areas after crossing the unprotected borders of neighboring Liberia and Sierra Leone. A major coordinated operation on the ground is needed to limit the geographic extension of the epidemic.

The causative agent of Ebola virus disease (EVD) is an RNA virus of the family *Filoviridae* and *genus Ebolavirus*. Five different *Ebolaviru*s strains have been identified, namely *Zaire ebolavirus* (EBOV), *Sudan ebolavirus* (SUDV), *Tai Forest ebolavirus* (TAFV), *Bundibugyo ebolavirus* (BDBV) and *Reston ebolavirus* (RESTV), with fruit bats considered as the most likely reservoir host [15]. The great majority of past Ebola outbreaks in humans have been linked to three Ebola strains: EBOV, SUDV and BDBV [16]. The Ebola virus, EBOV, (formerly designated *Zaire ebolavirus*), the deadliest of the five *Ebolaviru*s strains, was first identified in 1976 in Zaire (now the Democratic Republic of Congo) and its name was derived from the Ebola River located near the source of the first outbreak. Past Ebola outbreaks have been reported on average every 1.5 years [17], with a total of 7 prior outbreaks generating over 100 reported cases [18]. A recent study has estimated 22 million people distributed in areas of Central and West Africa to be at risk of Ebola [19].

Ebola is characterized by a high case fatality ratio which was nearly 90% in a past outbreak [20]. After an incubation period mostly ranging from 2 to 21 days, nonspecific symptoms appear, including sudden onset of fever, weakness, vomiting, diarrhea, headache and a sore throat. A fraction of patients may later develop severe internal and external hemorrhagic manifestations and experience multiple organ failures [21]. Except for RESTV, all other Ebola strains are pathogenic to humans. Human outbreaks may stem from direct human exposure to fruit bats or intermediate infected hosts that primarily comprise non-human primates (that is, gorillas, chimpanzees and monkeys). Human epidemics subsequently take off by direct human-to-human contact via bodily fluids or indirect contact with contaminated surfaces. Hence, stopping Ebola transmission should be feasible when the cases are detected early and managed properly, because this virus is not transmitted through the air or water [22]. Nevertheless, Ebola has been shown to spread through the air under carefully controlled laboratory conditions [23]. Hence, amplification of human-to-human transmission can result in the presence of suboptimal infection control measures in healthcare settings [24-26]. Unsafe burials that involve direct contact with Ebola-infected bodies also pose a major infection risk [20].

A review of key epidemiological parameters of EVD and our current understanding of the transmission dynamics and the effect of basic control interventions against this disease would be useful for guiding and assessing the potential effectiveness of control interventions during Ebola outbreaks. Specifically, here we review epidemiological data from past Ebola outbreaks including the basic reproduction number, the serial interval and the case fatality ratio. Subsequently, we carry out a comparative review of mathematical models of the spread and control of Ebola in the context of past and the ongoing epidemic in West Africa. We show that mathematical modeling offers useful insights into the risk of a major epidemic of EVD and the assessment of the impact of basic public health measures on disease spread. We illustrate the effects of demographic characteristics, such as the effective population size, size of spillover event (for example, details of initial conditions), baseline infection control measures in health care settings, and the timing of initiation of control interventions including enhancing the effectiveness of isolating infectious individuals, contact tracing to bring infectious individuals into isolation and social distancing interventions in the community.

### Natural history parameters of EVD

Due to the relatively few past Ebola outbreaks, available epidemiological data to infer the natural history parameters of EVD remain limited. Moreover, past outbreaks have been caused by different virus strains, making it difficult to judge whether a certain observed epidemiological characteristic is unique to the causative strain. Here, we extract published evidence and review Ebola epidemiological parameters from the literature, integrating estimates of the basic reproduction number, the asymptomatic ratio, the incubation period, the latent period, the symptomatic period, the infectious period, the serial interval and the case fatality ratio.

#### The basic reproduction number, R_0_

The basic reproduction number, *R*_0_, is interpreted as the average number of secondary cases caused by a typical infected individual throughout its entire course of infection in a completely susceptible population and in the absence of control interventions [27,28]. In the context of a partially susceptible population owing to prior exposure or vaccination, the (effective) reproduction number, *R*, quantifies the potential for infectious disease transmission. If *R* <1, transmission chains are not self-sustaining and are unable to generate a major epidemic. By contrast, an epidemic is likely to occur whenever *R* >1. When measured over time *t*, the effective reproduction number *R*_*t*_, can be helpful to quantify the time-dependent transmission potential and evaluate the effect of control interventions in almost ‘real time’ [29]. In summary, *R*_0_ is regarded as a summary measure of the transmissibility of infectious diseases, playing a key role in determining the required control effort (for example, intensity of quarantine and isolation strategies). *R*_0_ could also be useful for guiding the numbers of antivirals and vaccines that would be needed to achieve control whenever these are available.

#### R_0_ estimates for prior Ebola outbreaks in Central Africa

*R*_0_ has been estimated for prior EVD outbreaks in Central Africa using mathematical modeling and epidemiological data for two Ebola outbreaks, namely the 1995 outbreak in Democratic Republic of Congo and the 2000 Uganda outbreak, respectively [30,31]. Unlike the ongoing epidemic in West Africa, past outbreaks in Central Africa have been confined to relatively rural and isolated areas without spreading to urban sectors which facilitated the effective implementation of control interventions. Using a homogenous mixing SEIR (Susceptible-Exposed-Infectious-Removed) model that accounted for a gradual decay in the transmission rate at the start of interventions, Chowell *et al*. [32] estimated *R*_0_ at 1.83 for Congo and 1.34 for Uganda. Using the same epidemic model but employing a Bayesian estimation method, Lekone and Finkenstadt [33] estimated slightly lower values at 1.33 to 1.35 for the outbreak in Uganda. Legrand *et al*. employed a different modeling approach [19]: while allowing for homogeneous mixing, the study took into account three different transmission settings, that is, transmissions in community, hospital settings and during funerals. *R*_0_ was estimated at 2.7 for Congo, 1995 and 2.7 for Uganda, 2000, but estimates showed substantial uncertainty. Transmission from burials alone accounted for 1.8 secondary transmissions in Congo while community transmission in Uganda accounted for 2.6 secondary transmissions. Variability in *R*_0_ estimates across studies can be attributed to differences in model structure and underlying assumptions.

#### An assessment of R_0_ based on the growth rate of the 2014 Ebola epidemic in West Africa

A quick look at the ongoing epidemic in West Africa without delving into a too detailed analysis permits us to grasp the level of *R*_0_ for the ongoing Ebola outbreak. Assuming that the early epidemic data in Sierra Leone and Liberia are sufficient to be characterized by exponential growth dynamics, with growth rate *r*, the incidence (that is, the number of new cases at calendar time *t*) is modeled as

$$ i(t)=k\; \exp (rt), $$

where *k* is a constant. As the observed data are cumulative *I*(*t*), we integrate the above equation from the starting time of exponential growth *t*_0_ to the latest time *t*, that is,

$$ I(t)=\frac{k}{r}\left[ \exp (rt)- \exp \left(r{t}_0\right)\right]. $$

**Figure 1:**
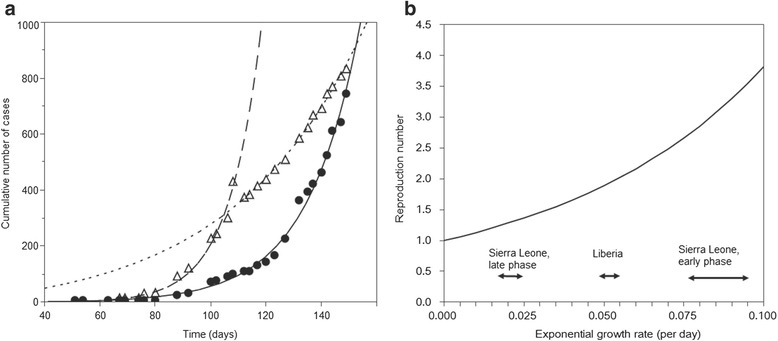
**Early transmission dynamics of Ebola virus disease (EVD) in Sierra Leone and Liberia, 2014. a)** The cumulative number of confirmed and probable cases of EVD as a function of calendar time [3]. Filled circles represent cases in Liberia, while unfilled triangles represent cases in Sierra Leone. The solid line shows the exponential growth fit to the incidence curve in Liberia. The dashed line is the exponential fit to the early phase in Sierra Leone (up to 8 July 2014), while the dotted line shows the exponential fit to the later phase in the same country. **b)** The relationship between the exponential growth rate and the corresponding reproduction number for EVD based on a Weibull distributed generation time with shape and scale parameters of 2.59 and 13.60, respectively. Arrows indicate the uncertainty range (95% confidence interval) of the exponential growth rate estimated from the corresponding epidemic data.

**Figure 2:**
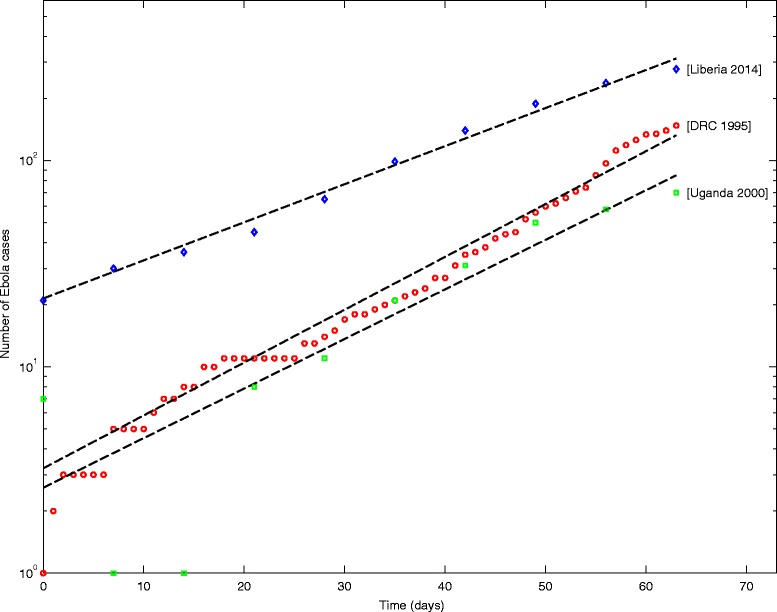
**Comparison of the growth trends for past outbreaks in Central Africa (Congo 1995 and Uganda 2000) with the ongoing Ebola epidemic in Liberia.** Time series of new Ebola case reports prior to the implementation of control interventions for the outbreak in Congo 1995 (9 May 1995) [24] and Uganda 2000 (22 October 2000) [100] and for the ongoing epidemic in Liberia from 15 June to 15 August 2014. Incidence data for the outbreaks in Central Africa are shown according to the dates of symptoms onset while the weekly incidence curve for the epidemic in Liberia comprises total cases based on the daily epidemic curve estimated in [7].

It should be noted that the cumulative number of cases does not follow a single exponential growth term. Assuming that the observed number of cases is Poisson distributed, the maximum likelihood estimate for *r* for Liberia is estimated at 0.053 (95% confidence interval (CI): 0.051, 0.055). The growth rate in Sierra Leone is largely divided into two phases with a greater growth rate in the early phase (which could reflect initial case clusters in hospital settings). Hence, *r* is estimated at 0.085 (95% CI: 0.080, 0.090) and 0.021 (95% CI: 0.019, 0.023) for the early and late phases, respectively (Figure 1a). Assuming that the mean generation time is 12 days (with standard deviation 5.2 days) based on contact tracing data from an outbreak in Uganda 2000 [34] (see below), *R*_0_ for Liberia is estimated at 1.96 (95% CI: 1.92, 2.01). For Sierra Leone, *R*_0_ is 3.07 (95% CI: 2.85, 3.32) and 1.30 (95% CI: 1.26, 1.33) for the early and late phases, respectively (Figure 1b). Estimates in Liberia and the late phase of Sierra Leone are roughly consistent with those published by Chowell *et al*. [30]. A comparison of the growth trends for past outbreaks in Central Africa (Congo 1995 and Uganda 2000) with the ongoing epidemic in Liberia is shown in Figure 2.

#### Mathematical modeling studies of the 2014 Ebola epidemic in West Africa

Recent studies have started to shed light on the transmission potential of the ongoing EVD epidemic. Specifically, three studies have estimated the basic reproduction number of EVD in the range of 1.5 to 2.5 [8,9,35]. Althaus [8] employed an SEIR model with the time-dependency of the reproduction number to capture effects of control interventions, following the model by Chowell *et al*. [18]; analyzing the country-specific data independently for each country, the estimates were 1.5 for Guinea, 2.5 for Sierra Leone and 1.6 for Liberia [8]. Gomes *et al*. [35] explicitly accounted for the risk of international spread, and the basic reproduction number ranged from 1.5 to 2.0. More importantly, this study employed a global epidemic model with mobility data, indicating that the short-term risk of international spread to outside Africa is small and that the expansion of the ongoing epidemic is more likely to occur in African countries [35]. Moreover, Fisman *et al*. estimated *R*_0_ at 1.8 using a two-parameter mathematical model that describes the epidemic growth and control [9].

Real-time estimation of the effective (time-dependent) reproduction number revealed estimates in line with *R*_0_ estimates derived from other studies. For instance, by measuring temporal variations in the epidemic growth rate during periods of epidemic growth, the reproduction number was approximated based on a classic formula of *R*_0_ for the SEIR model, which provided estimates in the range of 1.4 to 1.9 [36]. A different modeling study accounted for both local transmission and transnational spread across severely affected countries using a multivariate renewal process model which allowed the derivation of global and country-specific estimates of the reproduction number [7]. This study indicated that the effective reproduction number R_t_ from June to August 2014 ranged from 1.4 to 1.7 in Sierra Leone and Liberia. Hence, control could be reached by halting over half of the secondary transmissions per primary case whenever the reproduction number is below 2 [7]. Moreover, it is worth noting that the exponential growth in Ebola incidence is placing great pressure on healthcare facilities, which could affect time- and space-dependent variations in transmission dynamics and the surveillance system [37]. The analysis of available data using mathematical modeling should, therefore, carefully assess the quality and consistency of the surveillance system employed to collect epidemiological data. Hence, mathematical models should ideally be tied to characteristics of the surveillance system as much as possible to avoid potential bias [38].

#### Comparing R_0_ with other infectious diseases

For comparison with other filoviruses, the *R*_0_ for the 2005 Marburg Fever Outbreak in Angola has been consistently estimated at 1.6 using two different statistical modeling approaches [39,40]. For comparison with other infectious diseases transmitted by direct contact, *R*_0_ has been estimated at 2.6 for an outbreak of acute hemorrhagic conjunctivitis in Mexico [41]. In contrast, for respiratory infections, the reproduction number has been estimated for the SARS outbreaks in 2003 in the range 2.2 to 3.7 based on fitting transmission models to the progression of weekly cases prior to the start of control interventions [42,43], in the range 1.2 to 1.6 for seasonal influenza [44], 1.4 to 5.2 for influenza pandemics [45-50], 15 for pertussis, 17 for measles [27] and 1.2 to 1.3 for meningococcal meningitis [51].

#### Asymptomatic infection and incubation period

**Figure 3:**
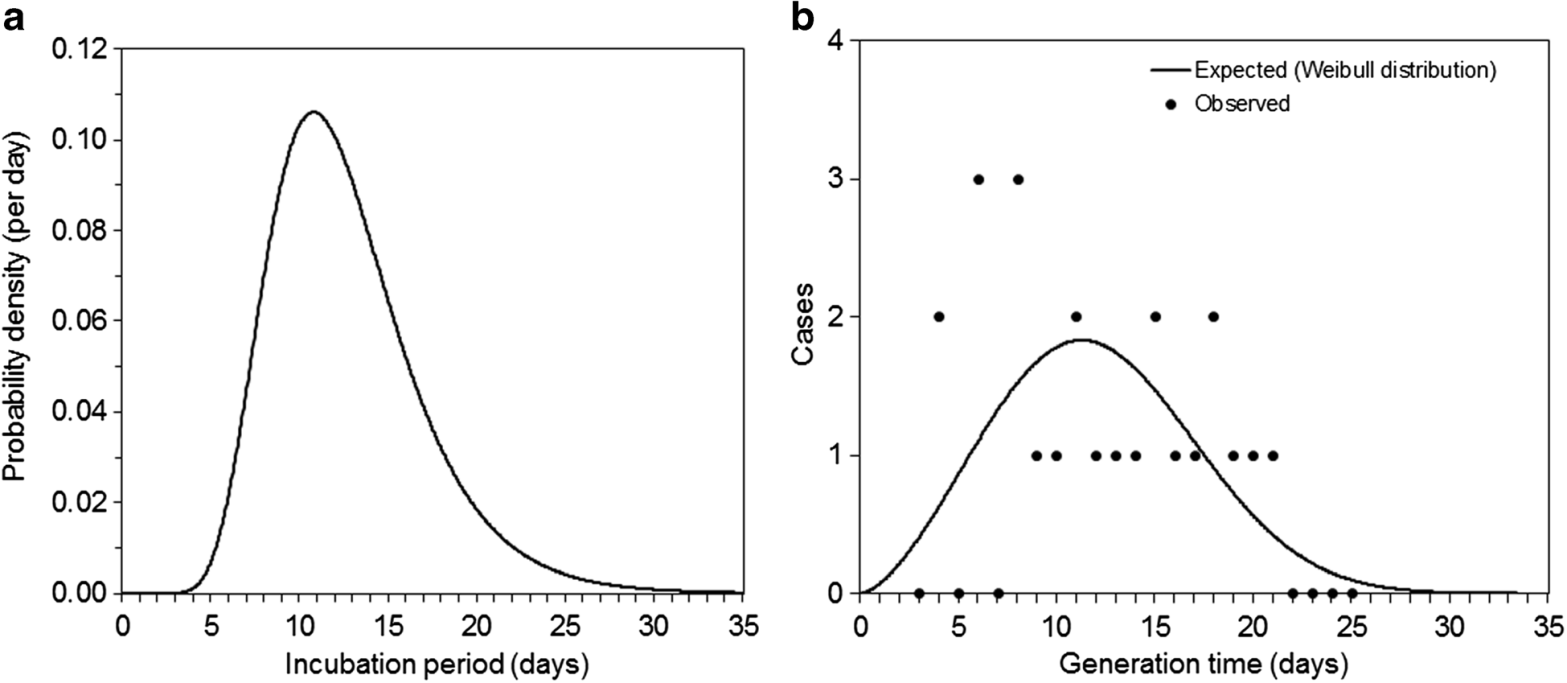
**Incubation period and generation time of Ebola virus disease (EVD). a)** The probability density function of the incubation period, that is, the time from infection to illness onset, fitted to a lognormal distribution is shown. The mean and the standard deviation are 12.7 and 4.3 days, respectively [54]. **b)** The generation time distribution, as collected from contact tracing data during the Ebola outbreak in Uganda, 2000, is fitted to a Weibull distribution. The mean and the standard deviation are 12.0 and 5.2 days, respectively.

Asymptomatic infection with Ebola virus is known to occur in a certain fraction of exposed individuals [52]. By analyzing the antibody responses among 24 asymptomatic close contacts of symptomatic patients, Leroy *et al*. found that 11 (45.8%) developed both immunoblobulin M (IgM) and IgG responses to Ebola antigens. However, the study subjects were only those who experienced close contacts, and an estimate of asymptomatic ratio for the general population was not obtained. The majority of cases developed illness 6 to 11 days after infection. A classical study of the Zaire strain [53] indicated that the mean incubation period, that is, the mean length of time from infection to illness onset, is 6.3 days with the 95% quantile 21 days. Reanalyzing the data set of household contacts during the Ebola outbreak in the Democratic Republic of Congo in 1995, Eichner *et al*. estimated the mean incubation period at 12.7 days (with standard deviation 4.31 days) [54]. The fitted lognormal distribution is redrawn in Figure 3a. By taking the 99 percentile point as the length of quarantine, Eichner *et al*. argues for movement restrictions of exposed healthy individuals for 25 days. Based on data for the first 9 months of the ongoing Ebola epidemic, a recent study estimated the mean incubation period at 11.4 days with no significant variation across the affected West African countries [6].

#### The serial interval

The serial interval defined as the time from illness onset in the primary case to illness onset in the secondary case [55], has been relatively well observed for EVD based on household or contact-tracing studies. A household study during the outbreak in DRC indicated that the minimum serial interval was 7 days, while the maximum was 17 days [56]. Findings based on contact tracing data for the outbreak in Uganda in 2000 were roughly consistent with those derived from household data [34]: mean (SD) and median (quartiles) estimates for the serial interval were 12.0 (5.2) and 11.5 (8 to 17) days, respectively. Figure 3b shows the serial-interval distribution along with a fitted Weibull distribution with scale and shape parameters estimated at 13.6 (95% CI: 11.4, 16.1) and 2.6 (95% CI: 1.8, 3.5), respectively. The Cramér-von Mises goodness-of-fit test did not reveal significant deviations between the observed data and fitted model distribution (W^2^ = 0.05, *P* =0.25). This estimate is in good agreement with that derived from data of the first 9 months of the ongoing epidemic in West Africa, which has been estimated at 15.3 ± (SD =9.3) days [6]. This distribution is key to quantifying the reproduction number using the exponential growth rate of cases during the early stage of an epidemic, because the conversion from the growth rate of cases to the reproduction number requires estimates of the generation time distribution [57] which is known to be informed by the serial interval and the incubation period [58].

#### The latent and infectious periods

Other parameters associated with the time course of EVD have not been rigorously ascertained. However, according to Bayesian model-based estimates from a past Ebola outbreak [33], the mean latent and infectious periods have been estimated at 9.4 and 5.7 days, respectively, using a vague prior and 10.1 and 6.5 days, respectively, for an informative prior. These exponential distributions based on a mathematical modeling study are the only available empirical evidence for these two time periods. The mean length of time from illness onset to death is approximately 10 days [24,56], but the transmissibility from the deceased from Ebola may account for a certain fraction of secondary transmissions [19]. Hence, the infectious period could be longer than the observable time to death if the burial is extended.

#### The case fatality ratio

The case fatality ratio (CFR) is calculated as the proportion of deaths among the total number of EVD cases, thereby informing the virulence of the infectious pathogen. EVD can be fatal, but it is important to note that the CFR being ‘almost 100%’ for EVD in general does not rest on any empirical arguments. For the well documented outbreaks of Ebola (excluding only isolated cases who are likely to have acquired infection from animal contact), the expected value of CFR has always been below 90% [31], with the range from 41% to 89%. The so-called Zaire strain is considered to be slightly more fatal than the Sudan strain. While the CFR for the Sudan strain ranges from 41% to 65%, the CFR for the Zaire strain ranges from 61% to 89%. Considering that the corresponding quartile for the Zaire strain, as determined by the distribution of outbreak-specific estimates, ranges from 73.3% to 84.3%, the CFR of the ongoing epidemic among cases with definitive recorded clinical outcomes for Guinea, Liberia and Sierra Leone has been consistently estimated at 70.8% (95% CI: 68.6 to 72.8), which is in good agreement with estimates from prior outbreaks. Nevertheless, it must be noted that earlier studies have not addressed ascertainment bias. It is important to follow up the reasons why the estimated 53% (as of 31 August 2014 which involved an underestimation bias due to time delay from illness onset to death) in real-time has been much lower than the published estimate of 70.8% among a portion of cases. Given the potential presence of asymptomatic cases, addressing ascertainment error may be the key to appropriately capture the disease burden for the entire population. Table 1 summarizes key epidemiological parameters for EVD.

**Table 1:** **Summary of empirical estimates of epidemiological parameters for Ebola virus disease (EVD)**

**Description**	**Value**	**Reference**
Incubation period	12.7 days (mean)	[54]
Latent period	10.1 days (mean)	[33]
Infectious period	6.5 days (mean)	[33]
Serial interval	12.0 days (mean)	[34]
Generation time	16.6 days (mean)	[34]
Time from illness onset to death	10 days (mean)	[24,56]
Case fatality ratio	41% to 65% (Sudan)	[31]
	61% to 89% (Zaire)	

### Models of Ebola transmission dynamics and control

The transmission dynamics of Ebola outbreaks in confined settings in Central Africa have been previously described using an SEIR epidemiological model [30] with the goal of quantifying the effects of social distancing interventions. In this model, the time-dependent transmission rate parameter *β*(*t*) captures the effects of implementing basic public health interventions over time. For instance, once interventions are put in place *τ* days after the onset of the outbreak, the time-dependent transmission rate could be modeled to shift from a ‘free course’ baseline value *β*_0_ to a value *β*_1_, where *β*_1_ < *β*_0_. More realistically, one can assume that the full effect of interventions is not seen immediately but gradually takes hold in the population, as modeled in [30]. In these models, the basic reproduction number, *R*_0_, in a completely susceptible population and in the absence of control interventions is computed as the product of the mean transmission rate during the intervention-free course of the outbreak, *β*_0_, and the mean infectious period, 1/*γ*_._ Hence, *R*_0_ is given by:

$$ {R}_0={\beta}_0/\gamma $$

More detailed epidemiological data and information about the contributions of different settings to transmission could guide the design of more elaborate models that could be helpful to quantify the effects of more specific intervention strategies. Legrand *et al*. [31] developed a structured transmission model to describe Ebola epidemics with contributions to the force of infection from the community, funerals and healthcare settings. The most distinctive feature of this model is that transmission during burial rituals is modeled by accounting for the duration of the burial and the intensity of transmission with infectious bodies. This model is comprised by six epidemiologically relevant states and thirteen parameters. The model was calibrated to data of the Ebola outbreaks in the Republic of Congo in 1995 and Uganda in 2000 by fitting three transmission rate parameters, one for each transmission setting and one parameter to quantify the effectiveness of interventions. The full model can be applied to the West African epidemic particularly for Guinea, Sierra Leone and Liberia where burial practices involve the touching of bodies of the deceased [59]. But this feature is believed to be less influential in transmission in the context of Nigeria where a limited outbreak developed. To illustrate the effects of control interventions during Ebola outbreaks, here we only account for transmission in the community and in healthcare settings by adjusting baseline transmission rates, diagnostic rates and enhancement of infection-control measures (for example, strict use of protective equipment by health-care workers and effective isolation of infectious individuals) (see for example, [27,28,42,43,60,61]). In this simpler setting, the population is divided into five categories: susceptible individuals (S); exposed individuals (E); infectious and symptomatic individuals (I); hospitalized individuals (H); and removed individuals after recovery or disease-induced death (R).

Susceptible individuals infected through contact with infectious individuals (secondary cases) enter the latent period at rate *β*(*t*) (*I* + *l*(*t*) *H*) /*N(t)* where *β*(*t*) is the mean human-to-human transmission rate per day, *l*(*t*) quantifies the relative transmissibility of hospitalized patients compared to symptomatic patients in the community, and *N*(*t*) is the total population size at time *t*. Thus, values of *l*(*t*) between 0 and 1 would reflect the effectiveness of hospital isolation measures that decrease Ebola transmission probability below that seen in the community, and values above 1.0 denote increased transmission in the hospital relative to the community, potentially due to biological and/or epidemiological reasons (for example, exposure to body fluids). Symptomatic infectious individuals *I* are hospitalized at the time-dependent average rate *γ*_a_(*t*) or recover without being hospitalized at the average rate *γ*_I._ Individuals in the ‘removed’ class do not contribute to the transmission process. For simplicity, one can assume that the time-dependent transmission rate *β*(*t*), relatively transmissibility of hospitalized patients, *l*(*t*), and the diagnostic rate *γ*_a_(*t*), remain constant values at *β*_0_, *l*_0_, and *γ*_a0_ prior to the implementation of comprehensive countermeasures. Hence, in this model the basic reproduction number, *R*_0_, is given by the following expression:

$$ {R}_0={\beta}_0\left[1/\left({\gamma}_{a0}+{\gamma}_I\right)+{l}_0\left(1/{\gamma}_r\right)\left({\gamma}_{a0}/\left({\gamma}_{a0}+{\gamma}_I\right)\right)\right]. $$

In this equation, (1/(*γ*_a0 +_*γ*_I_) is the mean infectious period of community cases, *γ*_a0_ /(*γ*_a0 +_*γ*_I_) is the fraction of symptomatic cases that are hospitalized, and 1/*γ*_r_ is the mean infectious period of hospitalized cases. This expression can be decomposed as the sum of the contributions of infectious individuals in the community and the hospital as follows:

$$ {R}_0 = {R}_{comm}+{R}_{hosp} $$

where *R*_comm_ = *β*_0_ /(*γ*_a0 +_*γ*_I_) and

*R*_hosp_ = *β*_0_*l*_0_ (1/*γ*_r_)(*γ*_a0_ /(*γ*_a0 +_*γ*_I_)).

**Figure 4:**
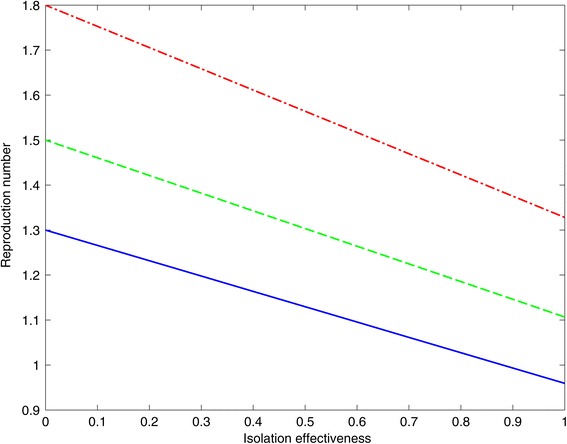
**The effects of isolation strategies on** ***R***_**0**_ **.** Basic reproduction number as a function of level of isolation effectiveness in health care settings for three different baseline values of *R*_0_: 1.3, 1.5 and 1.8. Epidemiological parameter values for EVD are shown in Table 1. The mean time from symptoms onset to diagnosis (*γ*_a0_) is assumed to be three days. The isolation effectiveness is given by 100*(1-*l*_0_) where *l*_0_ is the relative infectiousness of infectious individuals in health care settings. Baseline values of R_0_ are calibrated by adjusting the transmission rate *β* to achieve a given R_0_. EVD, Ebola virus disease; R_0_, basic reproduction number. Three lines represent results for three baseline values of *R*_0_: 1.3, 1.5 and 1.8.

**Figure 5:**
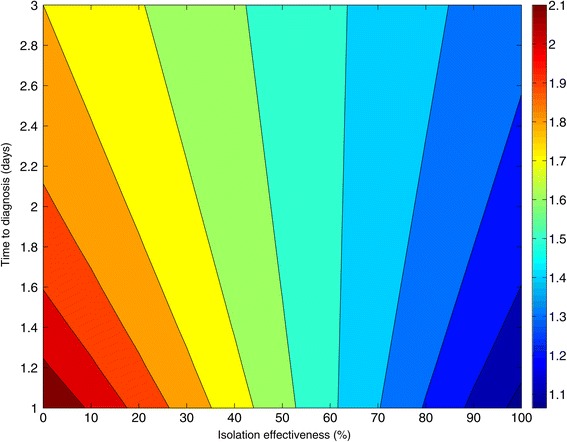
**The effects of isolation strategies and diagnostic rate on** ***R***_**0**_ **.** Basic reproduction number as a function of the combined effect of the level of isolation effectiveness and the diagnostic rate. Epidemiological mean parameter values for EVD are shown in Table 1. The mean time from symptoms onset to diagnosis (*γ*_a0_) is varied from one to three days. The isolation effectiveness is given by 100*(1-*l*_0_) where *l*_0_ is the relative infectiousness of infectious individuals in health care settings. The baseline value of R_0_ is set at 1.8. EVD, Ebola virus disease; R_0_, basic reproduction number.

Importantly, the above components for the reproduction number underscore the fact that the actual reproduction number could vary across regions as a function of the local capacity public health context (for example, infection control practices and availability of personal protective equipment for health care workers) and any local cultural practices that may influence transmission (for example, funeral traditions). Consequently, an outbreak may be very unlikely to unfold in developed countries simply as a result of baseline infection control measures in place (that is, *R*_0_ < 1) whereas poor countries with extremely weak or absent public health systems may be unable to control an Ebola outbreak (that is, *R*_0_ > 1). This suggests that local socioeconomic and sociocultural conditions are key determinants of disease spread, particularly in the context of the transmission dynamics of EVD. The impact of infection-control measures in health care settings is illustrated in Figure 4 for different initial values of baseline *R*_0_. The combined effect of the effectiveness of isolation measures and the diagnostic rate of symptomatic individuals on *R*_0_ is given in Figure 5.

### Initial transmission dynamics

The natural reservoir hosts of the Ebola virus have yet to be confirmed [62,63], but laboratory studies point to fruit bats as the most likely culprit harboring the Ebola virus in the natural habitat [63-66]. Ebola outbreaks among humans have been associated with direct exposure to fruit bats and mortality among other wild animals, which tend to succumb to the infection [67-69]. Epidemiological data support the notion that spillover events of Ebola virus from a natural reservoir (that is, fruit bats) or an intermediate host, such as non-human primates, into human populations occur with a certain frequency (for example, [70,71]), but only a small number of those introductions are ever correctly diagnosed and reported or successfully unfold human-to-human transmission chains that lead to outbreaks. This hinders our understanding of the frequency of spillover events as a function of time (for example, season) and its relationship with variation in climatological or socioeconomic variables. We note that two studies have associated the onset of Ebola outbreaks with climatological variables [72,73]. Specifically, Pinzon *et al*. reported evidence that Ebola outbreaks are correlated with drastic shifts from dry to wet conditions [72] while a more recent study by Ng *et al*. found lower temperature and higher absolute humidity associated with the onset of EVD outbreaks during 1976 to 2014 [73].

**Figure 6:**
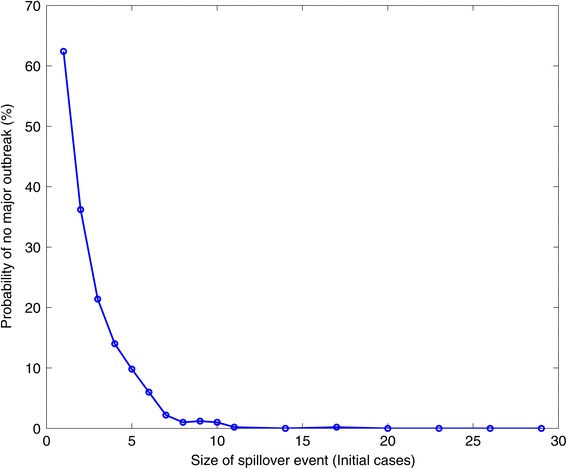
**The effects of size of spillover event on the likelihood of observing an outbreak.** Probability that no major outbreak unfolds as a function of the initial number of infectious cases introduced into the population. Epidemiological parameter values for EVD are shown in Table 1. The mean time from symptoms onset to diagnosis (*γ*_a0_) is set at three days. The isolation effectiveness is set at 0 (that is, *l*_0_. =1). Population size N is set at 100,000. The baseline value of R_0_ is set at 1.8. The curve corresponds to the mean of the results obtained from 500 model simulations. EVD, Ebola virus disease; R_0_, basic reproduction number.

In the context of the ongoing Ebola epidemic in West Africa, a recent study suggests that people in Sierra Leone have been previously exposed to the Ebola virus, but those introductions have not sparked major epidemics [2,71]. Moreover, the ongoing epidemic may have been triggered by a single spillover event as suggested by limited epidemiological data indicating that chains of transmission of reported cases can be traced back to one or two individuals [74]. This may be explained by the fact that Ebola introductions have historically tended to occur in remote, rural areas with sparse population structures characterized by higher disease extinction rates [75,76]. By contrast, the unprecedented size of the ongoing epidemic could have benefited from high population mobility across invisible borders, super spreading events [2] and secondary transmissions linked to health care settings [77]. Figure 6 illustrates the role of the size of spillover events (for example, the number of infectious cases initially introduced in the population) in triggering Ebola epidemics in naive populations by showing that the probability that a major epidemic occurs rapidly increases as a function of the initial number of Ebola cases. For instance, single-case introductions go extinct without developing into epidemics more than 60% of the time while five-case introductions lead to major epidemics more than 90% of the time.

### Delays in outbreak detection

**Figure 7:**
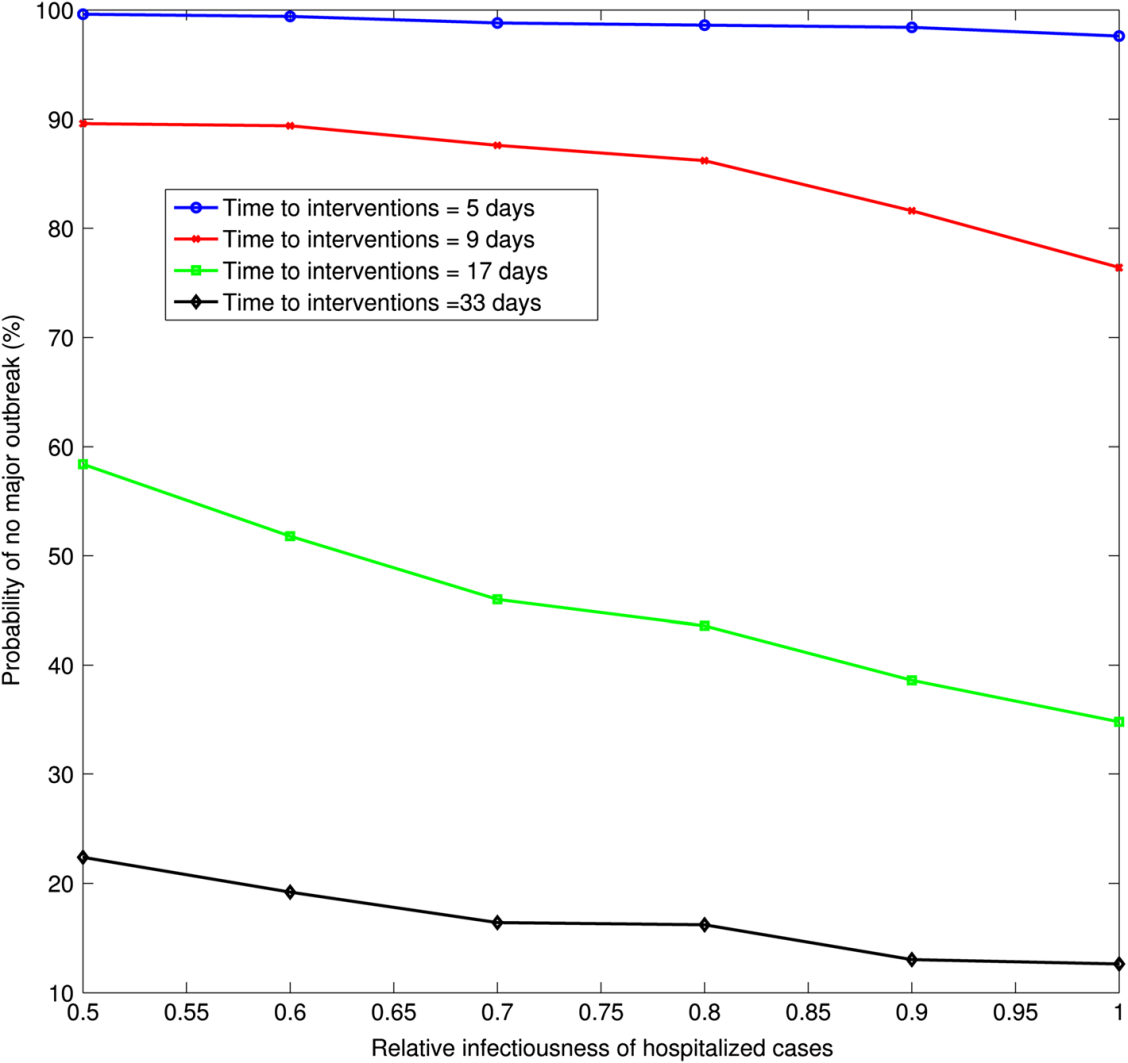
**The effects of size of baseline isolation effectiveness and timing of control interventions on the likelihood of observing an outbreak.** Probability that no major epidemic unfolds as a function of isolation effectiveness and timing of implementation of control interventions. Epidemiological parameter values for EVD are shown in Table 1. The mean time from symptoms onset to diagnosis (*γ*_a0_) is set at three days. The relative infectiousness of hospitalized cases is given by *l*_0_. Population size N is set at 100,000. The baseline value of R_0_ is set at 1.8 by adjusting the transmission rate. After the start of interventions, the transmission rate is reduced by 80% and the relative infectiousness of hospitalized individuals is reduced by 95% (that is, *l*_0_ = 1, *l*_1_ = 0.05). The curves shown correspond to the mean of the results obtained from 500 model simulations. EVD, Ebola virus disease; R_0_, basic reproduction number.

Several factors hamper the timely identification of Ebola outbreaks in Africa. First, only a small number of Ebola outbreaks have occurred in East and Central Africa since the first identified outbreak in 1976 relative to the regional burden of other endemic infectious diseases, such as malaria. Moreover, some areas at risk of Ebola have yet to experience Ebola outbreaks, which severely limits community-level knowledge of the disease. For instance, the ongoing 2014 epidemic of EVOB is reportedly the first to occur in West Africa [10]. Second, early symptoms of Ebola virus disease tend to be nonspecific (for example, many cases are only febrile) [24], which increases the likelihood of misdiagnosing Ebola with malaria or other locally endemic infectious diseases [13]. Unsuccessful treatment of febrile patients and/or the appearance of more specific symptoms during the course of the disease (for example, hemorrhagic manifestations) could increase the likelihood of an ‘astute’ public-health worker suspecting Ebola or other viral hemorrhagic fever [78]. Third, lack of epidemiological surveillance systems and diagnostic testing in poor countries further exacerbates the delay in detecting outbreaks. Consequently, the implementation of public health interventions may not start until case or death clusters start to be detected and investigated in the community by public health authorities. In general, the longer the delay in the implementation of control interventions, the higher the chances that the virus percolates from remote and sparsely populated areas into areas of high population density. The probability of observing major Ebola outbreaks is highly sensitive to the timing of initiation of control interventions as illustrated in Figure 7. This figure shows that a five-day delay is highly unlikely to result in major Ebola outbreaks. By contrast, more significant delays exceeding two weeks are likely to lead to Ebola outbreaks (Figure 7).

### Lack of public health infrastructure

**Figure 8:**
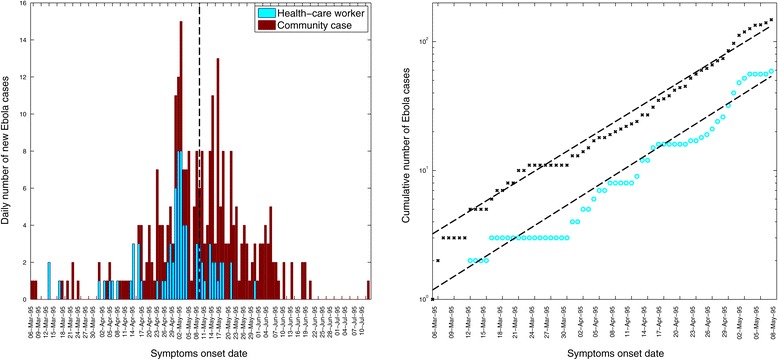
**The impact of Ebola on health care workers during the 1995 Ebola outbreak in The Republic of Congo.** Stacked bar plot of the epidemic curve of the 1995 Ebola outbreak in Republic of Congo to show the contributions of community and health-care worker cases. (left) Remarkably, the number of health care workers affected reached about 27% of the total number of reported Ebola cases. The vertical dashed line indicates the start of control interventions. The cumulative numbers of total cases (black stars) and of health care workers (blue circles) in logarithmic scale reveal a similar growth rate for both epidemic curves (right). Data were adapted from [24].

**Figure 9:**
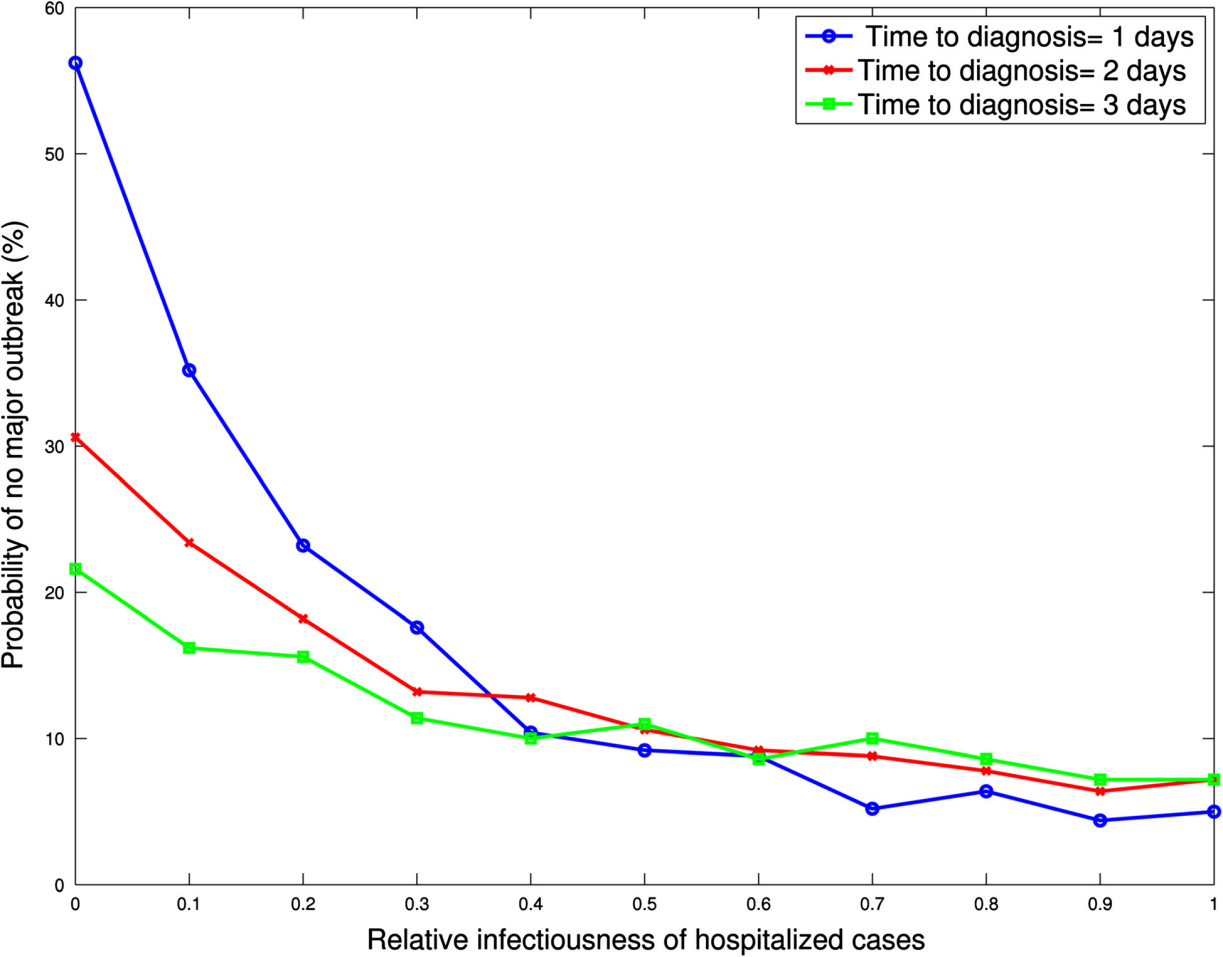
**The effects of size of baseline isolation effectiveness and diagnostic rate on the likelihood of observing an outbreak.** Probability that no major epidemic unfolds as a function of isolation effectiveness and time from symptoms onset to diagnosis. Epidemiological parameter values for EVD are shown in Table 1. The mean time from symptoms onset to diagnosis (*γ*_a0_) is set at one, two and three days. The relative infectiousness of hospitalized cases (*l*_0_) is varied from 0 to 1. Population size N is set at 100,000. The baseline value of R_0_ is set at 1.8 by adjusting the transmission rate. The curves shown correspond to the mean of the results obtained from 500 model simulations. EVD, Ebola virus disease; R_0_, basic reproduction number.

Basic infection control measures in health care settings are essential to avoid further spread of the disease to other patients, health care workers and visitors. Unfortunately, under-resourced African regions not only suffer from a critically low ratio of health-care workers to total population, but also lack essential personal protective equipment (PPE) (for example, gloves, gowns, face masks) to practice standard infection control measures. They also often lack the infrastructure and local capacity necessary to effectively trace contacts and isolate infectious individuals. Consequently, it is not surprising that Ebola outbreaks have been amplified in health care settings [24,25,79,80] including the ongoing epidemic in West Africa. Indeed, a total of 375 health care workers have developed EVD as of 23 September 2014 [81]. Fortunately, past experience also indicates that early and drastic enhancement of infection control measures in health care settings can substantially reduce the size and geographic scope of Ebola outbreaks [82,83]. For instance, Figure 8 shows that the rising trend in infected health care workers during the1995 Ebola outbreak in Congo rapidly declined following the implementation of control interventions. The combined impact of the rate of diagnosing symptomatic cases and the relative infectiousness of hospitalized cases on the probability of observing major epidemics is illustrated in Figure 9.

### Socio-cultural factors

Socio-cultural factors have not only contributed significantly to Ebola spread, but have also complicated the implementation of control interventions. Specifically, cultural practices involving touching the body of the deceased naturally (and greatly) contribute to the dissemination of the Ebola virus [59]. In particular, the potential for transmission to neighboring and distant areas by exposed funeral attendants could facilitate the development of major epidemics [1,31]. Moreover, the lack of prior experience or knowledge of the disease can lead communities to deny its existence and to associate illness with witchcraft or conspiracy theories presumably created by governments to gain control of populations or attract resources from the international community [77,80]. For instance, during the ongoing epidemic in West Africa, a group of individuals looted equipment and potentially contaminated materials in an isolation facility in a quarantined neighborhood [84]. Finally, the stigma carried by Ebola survivors and family members of Ebola victims could exacerbate disease spread. In particular, uninformed families tend to hide relatives and friends infected with Ebola to avoid being shunned by their own communities, which enhances transmission rates [85]. The problem is compounded by the high case fatality ratio of EVD whereby misinformed communities tend to associate case isolation with a death sentence.

### Future directions and conclusions

The ongoing epidemic in West Africa offers a unique opportunity to improve our current understanding of the transmission characteristics of EVD in humans, including the duration of immunity among Ebola survivors and the case fatality ratio in the presence or absence of supportive therapy [86,87], as well as the effectiveness of various control interventions [37]. For this purpose, there is a critical need to collect detailed epidemiological data in real-time during the ongoing epidemic through the establishment of efficient epidemiological surveillance systems in the affected areas. In addition, we cannot overemphasize the importance of collecting data relating to population behaviors influencing disease spread and control and how these have changed over time. It would also be important to record the level of adoption of preventive and social distancing measures in the community and adherence to infection control measures in health care settings. Detailed data regarding control interventions would also be critical to assess their effectiveness in reducing secondary transmissions including information on the changing numbers of isolation and treatment centers, healthcare workers, intensity of contact tracing activities and awareness campaigns in the community.

There is a scarcity of empirical studies quantifying transmission and the effects of control interventions implemented during past Ebola outbreaks [30,31]. Further work is also needed to quantify the effects of various interventions put in place during the ongoing epidemic in West Africa. Specifically, careful mathematical and statistical modeling studies could help ascertain the role of social distancing interventions (for example, school closures and cancellation of mass gathering events), infection control measures in health care settings (for example, isolation and other infection control measures among health care workers) and contact tracing and quarantine efforts [42,43,61,88-92]. In addition to individual epidemiological data, the timing of such interventions should be recorded along with the scale and extent of interventions (for example, closure of class rooms or entire schools). Intervention studies could reveal, for instance, whether effective infection control mechanisms in hospital settings could suffice to bring an epidemic under control or whether a combination of control strategies would be critical to ensure epidemic control (for example, *R* <1).

While a significant number of computational models have been developed to inform preparedness plans against pandemic influenza [93-95], comprehensive modeling studies to examine the spread and control of viral hemorrhagic fevers, including Ebola, in the context of the highly heterogeneous economic reality of African countries are yet to be developed. The shortage of modeling efforts could be explained by the fact that large Ebola outbreaks affecting large population settings were largely unexpected until now. To start filling this gap, datasets comprising detailed demographic, socio-economic, contact rates and population mobility estimates in the region (for example, commuting networks, air traffic) need to be integrated. Given that the disease is highly fatal, dynamic features of contact and mobility should also be closely investigated. Modeling studies with local demographic characteristics and human movement could be useful not only to assess the likelihood of major epidemics and carry out sensible projections on epidemic outcomes, but also to guide control efforts in the field, such as the estimation of the number, size and location of isolation facilities, the number of health workers and staff and essential supplies that would be needed to respond to a particular outbreak scenario as well as to quantify the effects of potential quarantine efforts in certain areas, border closures and air travel restrictions.

Proven treatments or vaccines against Ebola are still not available. Hence, our current working toolbox available to control the spread of Ebola still hinges on supportive medical care to increase the survival of those infected and basic non-pharmaceutical public health measures [96] to prevent transmission, namely: 1) infection control measures including standard precautions in health care settings; 2) rapid contact tracing and isolation of infectious individuals; and 3) social distancing interventions in the community which may include the dissemination of awareness campaigns to inform the population on how to avoid contracting the disease, quarantining individuals potentially exposed to infectious individuals and restricting the movement of communities exhibiting local transmission to prevent onward transmission. These actions must be conducted in close collaboration with local community leaders to effectively reach the population at large. With the ongoing epidemic in West Africa, the development of treatments and vaccines against Ebola is accelerating [96,97]. For instance, emergency use of a trickle of doses of an experimental drug with unknown efficacy or safety record in humans has been initiated during the outbreak [97]. Recent experiments in monkeys provide promising evidence that this experimental drug could have a significant impact on mortality burden during Ebola outbreaks [98]. Furthermore, a promising bivalent Ebola vaccine against the Zaire and Sudan Ebola strains is entering human safety trials in September 2014 [99] with an initial goal of building a stockpile of 10,000 doses by November 2014. Nevertheless, apart from pharmaceutical effects on the prognosis of infection, we have yet to examine how medication changes the transmission dynamics. Hence, careful studies could be useful for assessing the impacts of treatment on contact, transmission and diagnosis as well as on the disease burden [100]. If an Ebola vaccine is developed successfully, one could assess the effectiveness of pre-emptive and reactive treatment and vaccination plans in the context of limited stockpiles. Finally, it is worth noting that our efforts to prepare against current and future infectious disease threats should also include potential deliberate attempts to trigger epidemics, which are largely unexpected events but could pose high impact on public health and global economic activities.

## References

[1] Baize S, Pannetier D, Oestereich L, Rieger T, Koivogui L, Magassouba N, Soropogui B, Sow MS, Keïta S, De Clerck H, Tiffany A, Dominguez G, Loua M, Traoré A, Kolié M, Malano ER, Heleze E, Bocquin A, Mély S, Raoul H, Caro V, Cadar D, Gabriel M, Pahlmann M, Tappe D, Schmidt-Chanasit J, Impouma B, Diallo AK, Formenty P, Van Herp M, *et al*: **Emergence of Zaire Ebola Virus Disease in Guinea - preliminary report.** *N Engl J Med*, in press. 10.1056/NEJMoa1404505 24738640

[2] Gire SK, Goba A, Andersen KG, Sealfon RS, Park DJ, Kanneh L, Jalloh S, Momoh M, Fullah M, Dudas G, Wohl S, Moses LM, Yozwiak NL, Winnicki S, Matranga CB, Malboeuf CM, Qu J, Gladden AD, Schaffner SF, Yang X, Jiang PP, Nekoui M, Colubri A, Coomber MR, Fonnie M, Moigboi A, Gbakie M, Kamara FK, Tucker V, Konuwa E (2014). Genomic surveillance elucidates Ebola virus origin and transmission during the 2014 outbreak. Science.

[3] World Health Organization: *Ebola Virus Disease, West Africa –U pdate on 27 July 2014.* 2014.

[4] *Ebola virus disease update - West Africa, 08 August 2014*. 2014.

[5] Meltzer MI, Atkins CY, Santibanez S, Knust B, Petersen BW, Ervin ED, Nichol ST, Damon IK, Washington ML (2014). Estimating the future number of cases in the ebola epidemic –- liberia and sierra leone, 2014–2015. MMWR Surveill Summ.

[6] WHO Ebola Response Team: **Ebola Virus Disease in West Africa - the first 9 months of the epidemic and forward projections.** *N Engl J Med*, in press. 10.1056/NEJMoa1411100 PMC4235004 25244186

[7] Nishiura H, Chowell G: **Early transmission dynamics of Ebola virus disease (EVD), West Africa, March to August 2014.** *Euro Surveill* 2014, **19**. pii: 20894. 10.2807/1560-7917.es2014.19.36.20894 25232919

[8] Althaus CL: **Estimating the reproduction number of Zaire ebolavirus (EBOV) during the 2014 outbreak in West Africa.** *PLOS Currents Outbreaks* 2014. 10.1371/currents.outbreaks.91afb5e0f279e7f29e7056095255b288 PMC4169395 25642364

[9] Fisman D, Khoo E, Tuite A: **Early epidemic dynamics of the West African 2014 Ebola outbreak: estimates derived with a simple two-parameter model.** *PLOS Currents Outbreaks* 2014. 10.1371/currents.outbreaks.89c0d3783f36958d96ebbae97348d571 PMC4169344 25642358

[10] Fauci AS (2014). Ebola - underscoring the global disparities in health care resources. N Engl J Med.

[11] Bausch DG, Schwarz L (2014). Outbreak of ebola virus disease in Guinea: where ecology meets economy. PLoS Negl Trop Dis.

[12] *What is Contact Tracing? Centers for Disease Control and Prevention.* accessed on September 18, 2014.

[13] Okeke IN (2011). Divining without Seeds: the Case for Strengthening Laboratory Medicine in Africa.

[14] Del Rio C, Mehta AK, Lyon Iii GM, Guarner J (2014). Ebola Hemorrhagic Fever in 2014: the tale of an evolving epidemic. Ann Intern Med.

[15] Leroy EM, Kumulungui B, Pourrut X, Rouquet P, Hassanin A, Yaba P, Délicat A, Paweska JT, Gonzalez JP, Swanepoel R (2005). Fruit bats as reservoirs of Ebola virus. Nature.

[16] Briand S, Bertherat E, Cox P, Formenty P, Kieny MP, Myhre JK, Roth C, Shindo N, Dye C (2014). The international Ebola emergency. N Engl J Med.

[17] House T, Inglis N, Ross JV, Wilson F, Suleman S, Edeghere O, Smith G, Olowokure B, Keeling MJ (2012). Estimation of outbreak severity and transmissibility: Influenza A(H1N1)pdm09 in households. BMC Med.

[18] Centers for Disease Control and Prevention (CDC): *Outbreaks Chronology: Ebola Hemorrhagic Fever.*

[19] Pigott DM, Golding N, Mylne A, Huang Z, Henry AJ, Weiss DJ, Brady OJ, Kraemer MU, Smith DL, Moyes CL, Bhatt S, Gething PW, Horby PW, Bogoch II, Brownstein JS, Mekaru SR, Tatem AJ, Khan K, Hay SI: **Mapping the zoonotic niche of Ebola virus disease in Africa.** *Elife* 2014, **3**. ᅟ doi:10.7554/eLife.04395. 10.7554/eLife.04395 PMC4166725 25201877

[20] The World Health Organization: *Ebola Virus Disease.*

[21] Bwaka MA, Bonnet MJ, Calain P, Colebunders R, De Roo A, Guimard Y, Katwiki KR, Kibadi K, Kipasa MA, Kuvula KJ, Mapanda BB, Massamba M, Mupapa KD, Muyembe-Tamfum JJ, Ndaberey E, Peters CJ, Rollin PE, Van den Enden E, Van den Enden E (1999). Ebola hemorrhagic fever in Kikwit, Democratic Republic of the Congo: clinical observations in 103 patients. J Infect Dis.

[22] Centers for Disease Control and Prevention: *Transmission of Ebola (Ebola Virus Disease).* 2014. accessed on September 18, 2014.

[23] Weingartl HM, Embury-Hyatt C, Nfon C, Leung A, Smith G, Kobinger G (2012). Transmission of Ebola virus from pigs to non-human primates. Sci Rep.

[24] Khan AS, Tshioko FK, Heymann DL, Le Guenno B, Nabeth P, Kerstiëns B, Fleerackers Y, Kilmarx PH, Rodier GR, Nkuku O, Rollin PE, Sanchez A, Zaki SR, Swanepoel R, Tomori O, Nichol ST, Peters CJ, Muyembe-Tamfum JJ, Ksiazek TG (1999). The reemergence of Ebola hemorrhagic fever, Democratic Republic of the Congo, 1995. Commission de Lutte contre les Epidemies a Kikwit. J Infect Dis.

[25] Baron RC, McCormick JB, Zubeir OA (1983). Ebola virus disease in southern Sudan: hospital dissemination and intrafamilial spread. Bull World Health Organ.

[26] Ftika L, Maltezou HC (2013). Viral haemorrhagic fevers in healthcare settings. J Hosp Infect.

[27] Anderson RM, May RM (1991). Infectious Diseases of Humans.

[28] Diekmann O, Heesterbeek J (2000). Mathematical Epidemiology of Infectious Diseases: Model Building.

[29] Wallinga J, Teunis P (2004). Different epidemic curves for severe acute respiratory syndrome reveal similar impacts of control measures. Am J Epidemiol.

[30] Chowell G, Hengartner NW, Castillo-Chavez C, Fenimore PW, Hyman JM (2004). The basic reproductive number of Ebola and the effects of public health measures: the cases of Congo and Uganda. J Theor Biol.

[31] Legrand J, Grais RF, Boelle PY, Valleron AJ, Flahault A (2007). Understanding the dynamics of Ebola epidemics. Epidemiol Infect.

[32] Chowell G, Hengartner NW, Castillo-Chavez C, Fenimore PW, Hyman JM (2004). The basic reproductive number of Ebola and the effects of public health measures: the cases of Congo and Uganda. J Theor Biol.

[33] Lekone PE, Finkenstadt BF (2006). Statistical inference in a stochastic epidemic SEIR model with control intervention: Ebola as a case study. Biometrics.

[34] Francesconi P, Yoti Z, Declich S, Onek PA, Fabiani M, Olango J, Andraghetti R, Rollin PE, Opira C, Greco D, Salmaso S (2003). Ebola hemorrhagic fever transmission and risk factors of contacts, Uganda. Emerg Infect Dis.

[35] Gomes MF, Piontti AP, Rossi L, Chao D, Longini I, Halloran ME, Vespignani A: **Assessing the international spreading risk associated with the 2014 West African Ebola outbreak***. PLOS Currents Outbreaks* 2014. Edition 1. doi:10.1371/currents.outbreaks.cd818f63d40e24aef769dda7df9e0da5. 10.1371/currents.outbreaks.cd818f63d40e24aef769dda7df9e0da5 PMC4169359 25642360

[36] Towers S, Patterson-Lomba O, Castillo-Chavez C: **Temporal variations in the effective reproduction number of the 2014 West Africa Ebola outbreak.** *PLOS Currents Outbreaks* 2014. Edition 1. doi:10.1371/currents.outbreaks.9e4c4294ec8ce1adad283172b16bc908 10.1371/currents.outbreaks.9e4c4294ec8ce1adad283172b16bc908 PMC4169299 25642357

[37] Plachouras D, Sudre B, Testa M, Robesyn E, Coulombier D: **Letter to the editor: Early transmission dynamics of Ebola virus disease (EVD), West Africa, March to August 2014.** *Euro Surveill* 2014, **19**. pii: 20907. 10.2807/1560-7917.es2014.19.37.20907 25259536

[38] Nishiura H, Chowell G: **Authors’ reply: Feedback from modelling to surveillance of Ebola virus disease.** *Euro Surveill* 2014, **19**. pii: 20908. 10.2807/1560-7917.es2014.19.37.20908 25259537

[39] Bettencourt LM, Chowell G, Hyman JM, Bettencourt LM, Castillo-Chavez C (2009). An ensemble trajectory method for real-time modeling and prediction of unfolding epidemics: analysis of the 2005 Marburg Fever outbreak in Angola. Mathematical and Statistical Estimation Approaches.

[40] Ajelli M, Merler S (2012). Transmission potential and design of adequate control measures for Marburg hemorrhagic fever. PLoS One.

[41] Chowell G, Shim E, Brauer F, Diaz-Duenas P, Hyman JM, Castillo-Chavez C (2006). Modelling the transmission dynamics of acute haemorrhagic conjunctivitis: application to the 2003 outbreak in Mexico. Stat Med.

[42] Lipsitch M, Cohen T, Cooper B, Robins JM, Ma S, James L, Gopalakrishna G, Chew SK, Tan CC, Samore MH, Fisman D, Murray M (2003). Transmission dynamics and control of severe acute respiratory syndrome. Science.

[43] Riley S, Fraser C, Donnelly CA, Ghani AC, Abu-Raddad LJ, Hedley AJ, Leung GM, Ho LM, Lam TH, Thach TQ, Chau P, Chan KP, Lo SV, Leung PY, Tsang T, Ho W, Lee KH, Lau EM, Ferguson NM, Anderson RM (2003). Transmission dynamics of the etiological agent of SARS in Hong Kong: impact of public health interventions. Science.

[44] Chowell G, Miller MA, Viboud C (2007). Seasonal influenza in the United States, France, and Australia: transmission and prospects for control. Epidemiol Infect.

[45] Viboud C, Tam T, Fleming D, Handel A, Miller MA, Simonsen L (2006). Transmissibility and mortality impact of epidemic and pandemic influenza, with emphasis on the unusually deadly 1951 epidemic. Vaccine.

[46] Andreasen V, Viboud C, Simonsen L (2008). Epidemiologic characterization of the 1918 influenza pandemic summer wave in Copenhagen: implications for pandemic control strategies. J Infect Dis.

[47] Chowell G, Ammon CE, Hengartner NW, Hyman JM (2006). Transmission dynamics of the great influenza pandemic of 1918 in Geneva, Switzerland: assessing the effects of hypothetical interventions. J Theor Biol.

[48] Chowell G, Nishiura H, Bettencourt LM (2007). Comparative estimation of the reproduction number for pandemic influenza from daily case notification data. J R Soc Interface.

[49] Mills CE, Robins JM, Lipsitch M (2004). Transmissibility of 1918 pandemic influenza. Nature.

[50] Nishiura H (2007). Time variations in the transmissibility of pandemic influenza in Prussia, Germany, from 1918–19. Theor Biol Med Model.

[51] Trotter CL, Gay NJ, Edmunds WJ (2005). Dynamic models of meningococcal carriage, disease, and the impact of serogroup C conjugate vaccination. Am J Epidemiol.

[52] Leroy EM, Baize S, Volchkov VE, Fisher-Hoch SP, Georges-Courbot MC, Lansoud-Soukate J, Capron M, Debré P, McCormick JB, Georges AJ (2000). Human asymptomatic Ebola infection and strong inflammatory response. Lancet.

[53] Breman JG (1978). The epidemiology of Ebola hemorrhagic fever in Zaire, 1976. Ebola virus haemorrhagic fever.

[54] Eichner M, Dowell SF, Firese N (2011). Incubation period of ebola hemorrhagic virus subtype zaire. Osong Public Health Res Perspect.

[55] Fine PE (2003). The interval between successive cases of an infectious disease. Am J Epidemiol.

[56] Dowell SF, Mukunu R, Ksiazek TG, Khan AS, Rollin PE, Peters CJ (1999). Transmission of Ebola hemorrhagic fever: a study of risk factors in family members, Kikwit, Democratic Republic of the Congo, 1995. Commission de Lutte contre les Epidemies a Kikwit. J Infect Dis.

[57] Wallinga J, Lipsitch M (2007). How generation intervals shape the relationship between growth rates and reproductive numbers. Proc Biol Sci.

[58] Klinkenberg D, Nishiura H (2011). The correlation between infectivity and incubation period of measles, estimated from households with two cases. J Theor Biol.

[59] Hewlett BS, Amola RP (2003). Cultural contexts of Ebola in northern Uganda. Emerg Infect Dis.

[60] Chowell G, Fenimore PW, Castillo-Garsow MA, Castillo-Chavez C (2003). SARS outbreaks in Ontario, Hong Kong and Singapore: the role of diagnosis and isolation as a control mechanism. J Theor Biol.

[61] Gumel AB, Ruan S, Day T, Watmough J, Brauer F, van den Driessche P, Gabrielson D, Bowman C, Alexander ME, Ardal S, Wu J, Sahai BM (2004). Modelling strategies for controlling SARS outbreaks. Proc Biol Sci.

[62] Breman JG, Johnson KM, van der Groen G, Robbins CB, Szczeniowski MV, Ruti K, Webb PA, Meier F, Heymann DL (1999). A search for Ebola virus in animals in the Democratic Republic of the Congo and Cameroon: ecologic, virologic, and serologic surveys, 1979–1980. Ebola Virus Study Teams. J Infect Dis.

[63] Olival KJ, Hayman DT (2014). Filoviruses in bats: current knowledge and future directions. Viruses.

[64] Swanepoel R, Smit SB, Rollin PE, Formenty P, Leman PA, Kemp A, Burt FJ, Grobbelaar AA, Croft J, Bausch DG, Zeller H, Leirs H, Braack LE, Libande ML, Zaki S, Nichol ST, Ksiazek TG, Paweska JT, International Scientific and Technical Committee for Marburg Hemorrhagic Fever Control in the Democratic Republic of Congo (2007). Studies of reservoir hosts for Marburg virus. Emerg Infect Dis.

[65] Hayman DT, Emmerich P, Yu M, Wang LF, Suu-Ire R, Fooks AR, Cunningham AA, Wood JL (2010). Long-term survival of an urban fruit bat seropositive for Ebola and Lagos bat viruses. PLoS One.

[66] Yuan J, Zhang Y, Li J, Zhang Y, Wang LF, Shi Z (2012). Serological evidence of ebolavirus infection in bats. China. Virol J.

[67] Lahm SA, Kombila M, Swanepoel R, Barnes RF (2007). Morbidity and mortality of wild animals in relation to outbreaks of Ebola haemorrhagic fever in Gabon, 1994–2003. Trans R Soc Trop Med Hyg.

[68] Leroy EM, Epelboin A, Mondonge V, Pourrut X, Gonzalez JP, Muyembe-Tamfum JJ, Formenty P (2009). Human Ebola outbreak resulting from direct exposure to fruit bats in Luebo, Democratic Republic of Congo, 2007. Vector Borne Zoonotic Dis.

[69] Walsh PD, Abernethy KA, Bermejo M, Beyers R, De Wachter P, Akou ME, Huijbregts B, Mambounga DI, Toham AK, Kilbourn AM, Lahm SA, Latour S, Maisels F, Mbina C, Mihindou Y, Obiang SN, Effa EN, Starkey MP, Telfer P, Thibault M, Tutin CE, White LJ, Wilkie DS (2003). Catastrophic ape decline in western equatorial Africa. Nature.

[70] Jezek Z, Szczeniowski MY, Muyembe-Tamfum JJ, McCormick JB, Heymann DL (1999). Ebola between outbreaks: intensified Ebola hemorrhagic fever surveillance in the Democratic Republic of the Congo, 1981–1985. J Infect Dis.

[71] Schoepp RJ, Rossi CA, Khan SH, Goba A, Fair JN (2014). Undiagnosed acute viral febrile illnesses, Sierra Leone. Emerg Infect Dis.

[72] Pinzon JE, Wilson JM, Tucker CJ, Arthur R, Jahrling PB, Formenty P (2004). Trigger events: enviroclimatic coupling of Ebola hemorrhagic fever outbreaks. Am J Trop Med Hyg.

[73] Ng S, Basta N, Cowling B: **Association between temperature, humidity and ebolavirus disease outbreaks in Africa, 1976 to 2014.** *Euro Surveill* 2014, **19**. pii: 20892. 10.2807/1560-7917.es2014.19.35.20892 25210981

[74] Doucleff M: *Could A 2-Year-Old Boy Be 'Patient Zero’ For The Ebola Outbreak?* National Public Radio. 25 August 2014, 6:29 PM ET.

[75] Chowell G, Nishiura H, Viboud C (2012). Modeling rapidly disseminating infectious disease during mass gatherings. BMC Med.

[76] Allen L (2003). An Introduction to Stochastic Processes with Applications to Biology.

[77] Chan M (2014). Ebola Virus Disease in West Africa - no early end to the outbreak. N Engl J Med.

[78] Cohen J (2004). Containing the threat–don’t forget Ebola. PLoS Med.

[79] Fisher-Hoch SP (2005). Lessons from nosocomial viral haemorrhagic fever outbreaks. Br Med Bull.

[80] Borchert M, Mutyaba I, Van Kerkhove MD, Lutwama J, Luwaga H, Bisoborwa G, Turyagaruka J, Pirard P, Ndayimirije N, Roddy P, Van Der Stuyft P (2011). Ebola haemorrhagic fever outbreak in Masindi District, Uganda: outbreak description and lessons learned. BMC Infect Dis.

[81] WHO: *Ebola Response Roadmap Update - 26 September 2014.*

[82] Onyango CO, Opoka ML, Ksiazek TG, Formenty P, Ahmed A, Tukei PM, Sang RC, Ofula VO, Konongoi SL, Coldren RL, Grein T, Legros D, Bell M, De Cock KM, Bellini WJ, Towner JS, Nichol ST, Rollin PE (2007). Laboratory diagnosis of Ebola hemorrhagic fever during an outbreak in Yambio, Sudan, 2004. J Infect Dis.

[83] Nkoghe D, Kone ML, Yada A, Leroy E (2011). A limited outbreak of Ebola haemorrhagic fever in Etoumbi, Republic of Congo, 2005. Trans R Soc Trop Med Hyg.

[84] Liberia: **Ebola fears rise as clinic is looted.*** The Washington Post.*

[85] Phillip A: **They survived Ebola. Now they are shunned.*** The Washington Post.*

[86] Lamontagne F, Clement C, Fletcher T, Jacob ST, Fischer WA 2nd, Fowler RA, M S Epi: **Doing today’s work superbly well - treating Ebola with current tools.** *N Engl J Med*, in press. 10.1056/NEJMp1411310 25251518

[87] Kucharski AJ, Edmunds WJ: **Case fatality rate for Ebola virus disease in West Africa.** *Lancet* 2014. doi:10.1016/S0140-6736(14)61706-2. 10.1016/S0140-6736(14)61706-2 25260235

[88] Chowell G, Castillo-Chavez C, Fenimore PW, Kribs-Zaleta CM, Arriola L, Hyman JM (2004). Model parameters and outbreak control for SARS. Emerg Infect Dis.

[89] Lloyd-Smith JO, Galvani AP, Getz WM (2003). Curtailing transmission of severe acute respiratory syndrome within a community and its hospital. Proc Biol Sci.

[90] Day T, Park A, Madras N, Gumel A, Wu J (2006). When is quarantine a useful control strategy for emerging infectious diseases?. Am J Epidemiol.

[91] Mubayi A, Zaleta CK, Martcheva M, Castillo-Chavez C (2010). A cost-based comparison of quarantine strategies for new emerging diseases. Math Biosci Eng.

[92] Wang W, Ruan S (2004). Simulating the SARS outbreak in Beijing with limited data. J Theor Biol.

[93] Ferguson NM, Cummings DA, Fraser C, Cajka JC, Cooley PC, Burke DS (2006). Strategies for mitigating an influenza pandemic. Nature.

[94] Balcan D, Hu H, Goncalves B, Bajardi P, Poletto C, Ramasco JJ, Paolotti D, Perra N, Tizzoni M, Van den Broeck W, Colizza V, Vespignani A (2009). Seasonal transmission potential and activity peaks of the new influenza A(H1N1): a Monte Carlo likelihood analysis based on human mobility. BMC Med.

[95] Halloran ME, Ferguson NM, Eubank S, Longini IM, Cummings DA, Lewis B, Xu S, Fraser C, Vullikanti A, Germann TC, Wagener D, Beckman R, Kadau K, Barrett C, Macken CA, Burke DS, Cooley P (2008). Modeling targeted layered containment of an influenza pandemic in the United States. Proc Natl Acad Sci U S A.

[96] Frieden TR, Damon I, Bell BP, Kenyon T, Nichol S (2014). Ebola 2014 - new challenges, new global response and responsibility. N Engl J Med.

[97] Goodman JL (2014). Studying “secret serums” - toward safe, effective Ebola treatments. N Engl J Med.

[98] Qiu X, Wong G, Audet J, Bello A, Fernando L, Alimonti JB, Fausther-Bovendo H, Wei H, Aviles J, Hiatt E, Johnson A, Morton J, Swope K, Bohorov O, Bohorova N, Goodman C, Kim D, Pauly MH, Velasco J, Pettitt J, Olinger GG, Whaley K, Xu B, Strong JE, Zeitlin L, Kobinger GP: **Reversion of advanced Ebola virus disease in nonhuman primates with ZMapp.** *Nature* 2014. doi:10.1038/nature13777. 10.1038/nature13777 PMC4214273 25171469

[99] Kroll D: **GSK/NIAID Ebola vaccines to enter US, UK human safety trials.** *Forbes* 2014. http://www.forbes.com/sites/davidkroll/2014/08/28/gsk-niaid-ebola-vaccine-to-enter-uk-human-safety-trials-broad-international-collaboration/.

[100] **Outbreak of Ebola hemorrhagic fever, Uganda, August 2000–January 2001.** *Wkly Epidemiol Record* 2001, **76:**41–48.

